# High-Resolution Audio with Inaudible High-Frequency Components Induces a Relaxed Attentional State without Conscious Awareness

**DOI:** 10.3389/fpsyg.2017.00093

**Published:** 2017-02-01

**Authors:** Ryuma Kuribayashi, Hiroshi Nittono

**Affiliations:** Graduate School of Human Sciences, Osaka UniversityOsaka, Japan

**Keywords:** high-resolution audio, electroencephalogram, alpha power, event-related potential, vigilance task, attention, conscious awareness, hypersonic effect

## Abstract

High-resolution audio has a higher sampling frequency and a greater bit depth than conventional low-resolution audio such as compact disks. The higher sampling frequency enables inaudible sound components (above 20 kHz) that are cut off in low-resolution audio to be reproduced. Previous studies of high-resolution audio have mainly focused on the effect of such high-frequency components. It is known that alpha-band power in a human electroencephalogram (EEG) is larger when the inaudible high-frequency components are present than when they are absent. Traditionally, alpha-band EEG activity has been associated with arousal level. However, no previous studies have explored whether sound sources with high-frequency components affect the arousal level of listeners. The present study examined this possibility by having 22 participants listen to two types of a 400-s musical excerpt of *French Suite No. 5* by J. S. Bach (on cembalo, 24-bit quantization, 192 kHz A/D sampling), with or without inaudible high-frequency components, while performing a visual vigilance task. High-alpha (10.5–13 Hz) and low-beta (13–20 Hz) EEG powers were larger for the excerpt with high-frequency components than for the excerpt without them. Reaction times and error rates did not change during the task and were not different between the excerpts. The amplitude of the P3 component elicited by target stimuli in the vigilance task increased in the second half of the listening period for the excerpt with high-frequency components, whereas no such P3 amplitude change was observed for the other excerpt without them. The participants did not distinguish between these excerpts in terms of sound quality. Only a subjective rating of inactive pleasantness after listening was higher for the excerpt with high-frequency components than for the other excerpt. The present study shows that high-resolution audio that retains high-frequency components has an advantage over similar and indistinguishable digital sound sources in which such components are artificially cut off, suggesting that high-resolution audio with inaudible high-frequency components induces a relaxed attentional state without conscious awareness.

## Introduction

High-resolution audio has recently emerged in the digital music market due to recent advances in information and communications technologies. Because of a higher sampling frequency and a greater bit depth than conventional low-resolution audio such as compact disks (CDs), it provides a closer replication of the real analog sound waves. Sampling frequency means the number of samples per second taken from a sound source through analog-to-digital conversion. Bit depth is the number of possible values in each sample and expressed as a power of two. A higher sampling frequency makes the digitization of sound more accurate in the time-frequency domain, whereas a greater bit depth increases the resolution of the sound. What kind of advantage does the latest digital audio have for human beings? This question has not been sufficiently discussed. The present investigation used physiological, behavioral, and subjective measures to provide evidence that high-resolution audio affects human psychophysiological state without conscious awareness.

The higher sampling frequency enables higher frequency sound components to be reproduced, because one-half of the sampling frequency defines the upper limit of reproducible frequencies (as dictated by the Nyquist–Shannon sampling theorem). However, in conventional digital audio, sampling frequency is usually restrained so that sounds above 20 kHz are cut off in order to reduce file sizes for convenience. This reduction is based on the knowledge that sounds above 20 kHz do not influence sound quality ratings (Muraoka et al., 1981) and do not appear to produce evoked brain magnetic field responses (Fujioka et al., 2002).

In contrast to this conventional digital recording process in which inaudible high-frequency components are cut off, high-resolution music that retains such components has been repeatedly shown to affect human electroencephalographic (EEG) activity (Oohashi et al., 2000, 2006; Yagi et al., 2003a; Fukushima et al., 2014; Kuribayashi et al., 2014; Ito et al., 2016). This effect is often called “hypersonic” effect. In these studies, only the presence or absence of inaudible high-frequency components is manipulated while the sampling frequency and the bit depth are held constant. Interestingly, this effect appears with a considerable delay (i.e., 100–200 s after the onset of music). However, it remains unclear what kind of psychological and cognitive states are associated with this effect. These studies also suggest that it is difficult to distinguish in a conscious sense between sounds with and without inaudible high-frequency components (full-range vs. high-cut). Some studies have shown that full-range audio is rated as better sound quality (e.g., a softer tone, more comfortable to the ears) than high-cut audio (Oohashi et al., 2000; Yagi et al., 2003a). Another study has shown that participants are not able to distinguish between the two types of digital audio, with no significant differences found for subjective ratings of sound qualities (Kuribayashi et al., 2014). The feasibility of discrimination seems to depend on the kinds of audio sources and individuals (Nishiguchi et al., 2009). Regarding behavioral aspects, it has been shown that people listen to full-range sounds at a higher level of sound volume than high-cut sounds (Yagi et al., 2003b, 2006; Oohashi et al., 2006).

Previous studies have examined the effect of inaudible high-frequency components on EEG activity while listening to music under resting conditions. It has been shown that EEG alpha-band (8–13 Hz) frequency power is greater for high-resolution music with high-frequency components than for the same sound sources without them (Oohashi et al., 2000, 2006; Yagi et al., 2003a). The effect appears more clearly in a higher part of the conventional alpha-band frequency of 8–13 Hz (10.5–13 Hz: Kuribayashi et al., 2014; 11–13 Hz: Ito et al., 2016). Ito et al. (2016) reported that low beta-band (14–20 Hz) EEG power also showed the same tendency to increase as high alpha-band EEG power.

A study using positron emission tomography (PET) revealed that the brainstem and thalamus areas were more activated when hearing full-range as compared with high-cut sounds (Oohashi et al., 2000). Because such activation may support a role of the thalamus in emotional experience (LeDoux, 1993; Vogt and Gabriel, 1993; Blood and Zatorre, 2001; Jeffries et al., 2003; Brown et al., 2004; Lupien et al., 2009) and also in filtering or gating sensory input (Andreasen et al., 1994), Oohashi et al. (2000) speculated that the presence of inaudible high-frequency components may affect the perception of sounds and some aspects of human behavior.

Another line of research suggests a link between cognitive function and alpha-band as well as beta-band EEG activities. Alpha-band EEG activity is thought to be associated not only with arousal and vigilance levels (Barry et al., 2007) but also with cognitive tasks involving perception, working memory, long-term memory, and attention (e.g., Basar, 1999; Klimesch, 1999; Ward, 2003; Klimesch et al., 2005). Higher alpha-band activity is considered to inhibit task-irrelevant brain regions so as to serve effective disengagement for optimal processing (Jensen and Mazaheri, 2010; Foxe and Snyder, 2011; Weisz et al., 2011; Klimesch, 2012; De Blasio et al., 2013).

Beta-band power is broadly thought to be associated with motor function when it is derived from motor areas (Hari and Salmelin, 1997; Crone et al., 1998; Pfurtscheller and Lopes da Silva, 1999; Pfurtscheller et al., 2003). Moreover, beta power has been shown to increase with corresponding increases in arousal and vigilance levels, which indicates that participants get engaged in a task (e.g., Sebastiani et al., 2003; Aftanas et al., 2006; Gola et al., 2012; Kamiñski et al., 2012).

What kind of advantage does high-resolution audio with inaudible high-frequency components have for human beings? What remains unclear is what kind of psychophysiological states high-resolution audio induces, along with the corresponding increase in alpha- and beta-band EEG activities. To monitor listeners’ arousal level, we asked participants to listen to a musical piece while performing a visual vigilance task that required sustained attention in order to continuously respond to specific stimuli. Two types of high-resolution audio of the same musical piece were presented using a double-blind method: With or without inaudible high-frequency components.

EEG was recorded along with other psychophysiological measures: Heart rate (HR), heart rate variability (HRV), and facial electromyograms (EMGs). The former two measures index autonomic nervous system activities. HRV contains two components with different frequency bands: High frequency (HF; 0.15-0.4 Hz), and low frequency (LF; 0.04-0.15 Hz). HF and LF activities are mediated by vagal and vagosympathetic activations, respectively (Malliani et al., 1991; Malliani and Montano, 2002). The LF/HF power ratio is sometimes used as an index parameter that shows the sympathetic activities. Facial EMGs in the regions of the corrugator supercilii and the zygomaticus major muscles have been used as indices of negative and positive affects, respectively (Larsen et al., 2003). Decrements in vigilance task performance such as longer reaction times (RTs) and higher error rates are interpreted as reflecting the decrease in arousal level, which is also reflected in the electrical activity of the brain (Fruhstorfer and Bergström, 1969). Besides ongoing EEG activity, event-related potentials (ERPs) are associated with vigilance task performance. When vigilance task performance decreases, the amplitude of P3, a positive ERP component observed dominantly at parietal recording sites between 300 and 600 ms after stimulus onset, decreases and its latency increases (Fruhstorfer and Bergström, 1969; Davies and Parasuraman, 1977; Parasuraman, 1983). The P3 outcomes are thought to be modulated not only by overall arousal level but also by attentional resource allocation (Polich, 2007). P3 amplitude has been shown to be larger when greater attentional resources are allocated to the eliciting stimulus. It is thus thought that P3 amplitude can serve as a measure of processing capacity and mental workload (Kok, 1997, 2001).

In the present study, physiological, behavioral, and subjective measures were recorded to examine what kind of advantage high-resolution audio with inaudible high-frequency components has. Specifically, we were interested in how the increase in alpha- and beta-band EEG activities is associated with listeners’ arousal and vigilance level. Using a double-blind method, two types of high-resolution audio of the same musical piece (with or without inaudible high-frequency components) were presented while participants performed a vigilance task in the visual modality.

## Materials and Methods

### Participants

Twenty-six student volunteers at Hiroshima University gave their informed consent and participated in the study. Four participants had to be excluded due to technical problems. The remaining 22 participants (14 women, 18–24 years, *M* = 20.6 years) did not report any known neurological dysfunction or hearing deficit. They were right-handed according to the Edinburgh Inventory (*M* = 84.1 ± 12.8). All reported to have correct or corrected-to-normal vision. Eight participants had the experience of learning musical instruments for a few years, but none of them were professional musicians. The Research Ethics Committee of the Graduate School of Integrated Arts and Sciences in Hiroshima University approved the experimental protocol.

### Stimuli and Task

The present study used the same materials that were used in Kuribayashi et al. (2014). The first 200-s portion of *French Suite No. 5* by J. S. Bach (on cembalo, 24-bit quantization, 192 kHz A/D sampling) was selected. In the present study, this portion was played twice to produce a 400-s excerpt. The original (full-range) excerpt is rich in high-frequency components. A high-cut version of the excerpt was produced by removing such components using a low-pass finite impulse response digital filter with a very steep slope (cutoff = 20 kHz, slope = –1,673 dB/oct). This linear-phase filter does not cause any phase distortion. Although the filter produces very small ripples (1.04^∗^10^-2^ dB), they are negligible and it is unlikely to affect auditory perception. Sounds were amplified using AI-501DA (TEAC Corporation, Tokyo, Japan) controlled by dedicated software on a laptop PC. Two loudspeakers with high-frequency tweeters (PM1; Bowers & Wilkins, Worthing, England) were located 1.5 m diagonally forward from the listening position. The sound pressure level was set at approximately 70 dB (A). Calibration measurements at the listening position ensured that the full-range excerpt contained abundant high-frequency components and that the high-frequency power of the high-cut excerpt (i.e., components over 20 kHz) did not differ from that of background noise. The average power spectra of the excerpts are available at http://links.lww.com/WNR/A279 as Supplemental Digital Content of Kuribayashi et al. (2014).

An equiprobable visual Go/NoGo task was conducted using a cathode ray tube (CRT) computer monitor (refresh rate = 100 Hz) in front of participants. A block consisted of 120 visual stimuli: 60 targets (either ‘T’ or ‘V’, 30 each) and 60 non-targets (‘O’) in a randomized order. The visual stimuli were 200 ms in duration and presented with a mean stimulus onset asynchrony (SOA) of 5 s (range = 3-7 s). Button-press responses with the left and right index fingers were required to ‘T’ and ‘V’ (or ‘V’ and ‘T’) respectively, as quickly and accurately as possible.

### Procedure

The study was conducted using a double-blind method. Participants listened to two versions of the 400-s musical excerpt (with or without high-frequency components) while performing the Go/NoGo task. Participants also performed the task under silent conditions for 100 s before and after music presentation (pre- and post-music periods). The presentation order of the two excerpts was counterbalanced across the participants. EEG, HR, and facial EMGs were recorded during task performance. After listening to each excerpt, participants completed a sound quality questionnaire consisting of 10 pairs of adjectives and then reported their mood states on the Affect Grid (Russell et al., 1989) and multiple mood scales (Terasaki et al., 1992). At the end of the experiment, participants judged which excerpt contained high-frequency components by making a binary choice between them.

### Physiological Recording

Psychophysiological measures were recorded with a sampling rate of 1000 Hz using QuickAmp (Brain Products, Gilching, Germany). Filter bandpass was DC to 200 Hz. EEG was recorded from 34 scalp electrodes (Fp1/2, Fz, F3/4, F7/8, FC1/2, FC5/6, FT9/10, Cz, T7/8, C3/4, CP1/2, CP5/6, TP9/10, Pz, P3/4, P7/8, PO9/10, Oz, O1/2) according to the extended 10–20 system. Four additional electrodes (supra-orbital and infra-orbital ridges of the right eye and outer canthi) were used to monitor eye movements and blinks. EEG data were recorded using the average reference online and re-referenced to the digitally linked earlobes (A1–A2) offline. EEG data were resampled at 250 Hz and were filtered offline (1–60 Hz band pass, 24 dB/oct for EEG analysis; 0.1–60 Hz band pass, 24 dB/oct for ERP analysis). Ocular artifacts were corrected using a semi-automatic independent component analysis method implemented on Brain Vision Analyzer 2.04 (Brain Products). The components that were easily identifiable as artifacts related to blinks and eye movements were removed.

Heart rate was measured by recording electrocardiograms from the left ankle and the right hand. The R–R intervals were calculated and converted into HR in bpm. For facial EMGs, electrical activities over the zygomaticus major and corrugator supercilii regions were recorded using bipolar electrodes affixed above the left brow and on the left cheek, respectively (Fridlund and Cacioppo, 1986). The EMG data were filtered offline (15 Hz high-pass, 12 dB/oct) and fully rectified (Larsen et al., 2003).

### Data Reduction and Statistical Analysis

A total of 600 s (including silent periods) was divided into six 100-s epochs. For EEG analysis, each 100-s epoch was divided into 97 2.048-s segments with 1.024 s overlap. Power spectrum was calculated by Fast Fourier Transform with a Hanning window. The total powers (μV^2^) of the following frequency bands were calculated: Delta (1–4 Hz), theta (4–8 Hz), low-alpha (8–10.5 Hz), high-alpha (10.5–13 Hz), low-beta (13–20 Hz), high-beta (20–30 Hz), and gamma (36–44 Hz). The square root of the total power (μV) was used for statistical analysis, following the procedure of previous studies (Oohashi et al., 2000, 2006; Kuribayashi et al., 2014). The scalp electrode sites were grouped into four regions: Anterior Left (AL: Fp1, F3, F7, FC1, FC5, FT9), Anterior Right (AR: Fp2, F4, F8, FC2, FC6, FT10), Posterior Left (PL: CP1, CP5, TP9, P3, P7, PO9, O1), and Posterior Right (PR: CP2, CP6, TP10, P4, P8, PO10, O2). For RT, EMG, and HR, the mean values of each 100-s epoch were calculated. Mean RT and EMG values were log-transformed before statistical analysis.

Heart rate variability analysis was done by using Kubios HRV 2.2 (Tarvainen et al., 2014). The last 300-s (5-min) epoch of the 400-s listening period was selected according to previously established guidelines (Berntson et al., 1997). Prior to spectrum estimation, the R–R interval series is converted to equidistantly sampled series via piecewise cubic spline interpolation. The spectrum is estimated using an autoregressive modeling based method. The total powers (ms^2^) were calculated for LF (0.04–0.15 Hz) and HF (0.15–0.4 Hz) bands, and LF/HF power ratio was obtained. The square roots of LF and HF (ms) were used for statistical analysis.

For ERP analysis, the total 400-s listening period was divided into two 200-s epochs, to secure a reasonable number of Go trials (around 20). Silent periods (pre- and post-music epoch) were not included in the calculation. Those trials found to have Go omissions, Go misses (incorrect hand response to ‘T’ or ‘V’), or NoGo responses (commission errors) were excluded from further processing steps. Go and NoGo responses were separately averaged to produce ERPs. Epochs (200 ms before stimulus presentation until 1000 ms after the presentation) were baseline corrected (-200 ms until 0 ms). The peak of a P3 wave was identified within a latency range of 350-500 ms at Pz where P3 amplitude is dominant topographically.

Each measure was subjected to a repeated measures analysis of variance (ANOVA) with sound type (full-range vs. high-cut) and epoch (pre-music, 0-100, 100-200, 200-300, 300-400 s, and post-music for EEG data; 0-200 and 200-400 s for ERP data) as factors. To compensate for possible type I error inflation by the violation of sphericity, multivariate ANOVA solutions are reported (Vasey and Thayer, 1987). The significance level was set at 0.05. For *post hoc* multiple comparisons of means, the comparison-wise level of significance was determined by the Bonferroni method.

## Results

### EEG Measures

**FIGURE 1:**
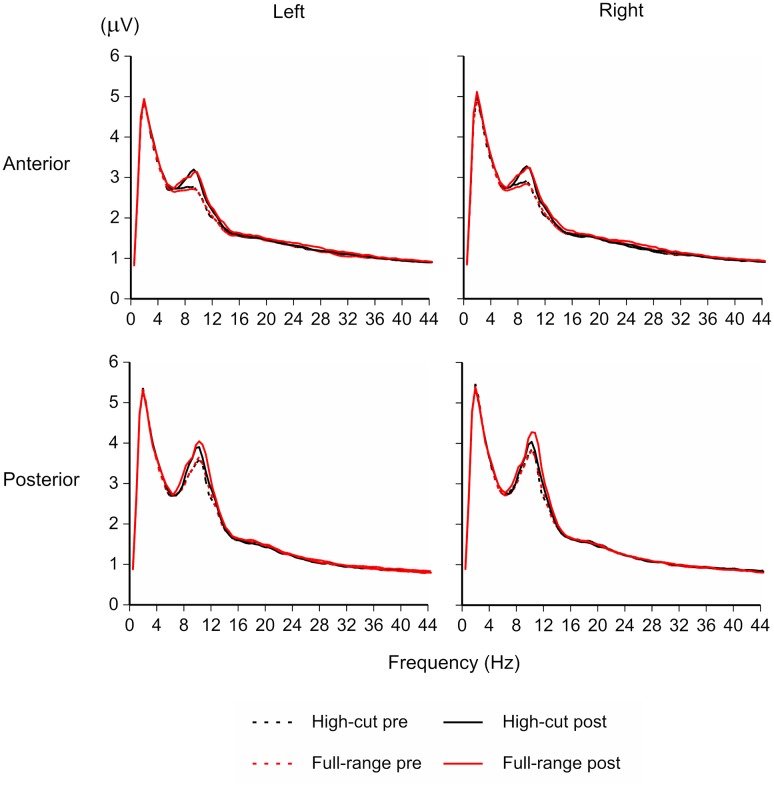
**EEG amplitude spectrogram for the full-range and high-cut conditions in the silent periods (100-s epochs before and after listening to music).** The amplitude of the peak around 10 Hz was increased after listening to music, in particular after listening to the full-range version of the sound source that contains high-frequency components.

**Figure 1** shows the EEG amplitude spectrogram for the four regions in the silent conditions (pre- and post-music periods). Although participants were performing a visual vigilance task with eyes opened, a peak around 10 Hz appears clearly. The amplitude of the peak appears to be increased after listening to music, in particular after listening to the full-range version of the musical piece.

**FIGURE 2:**
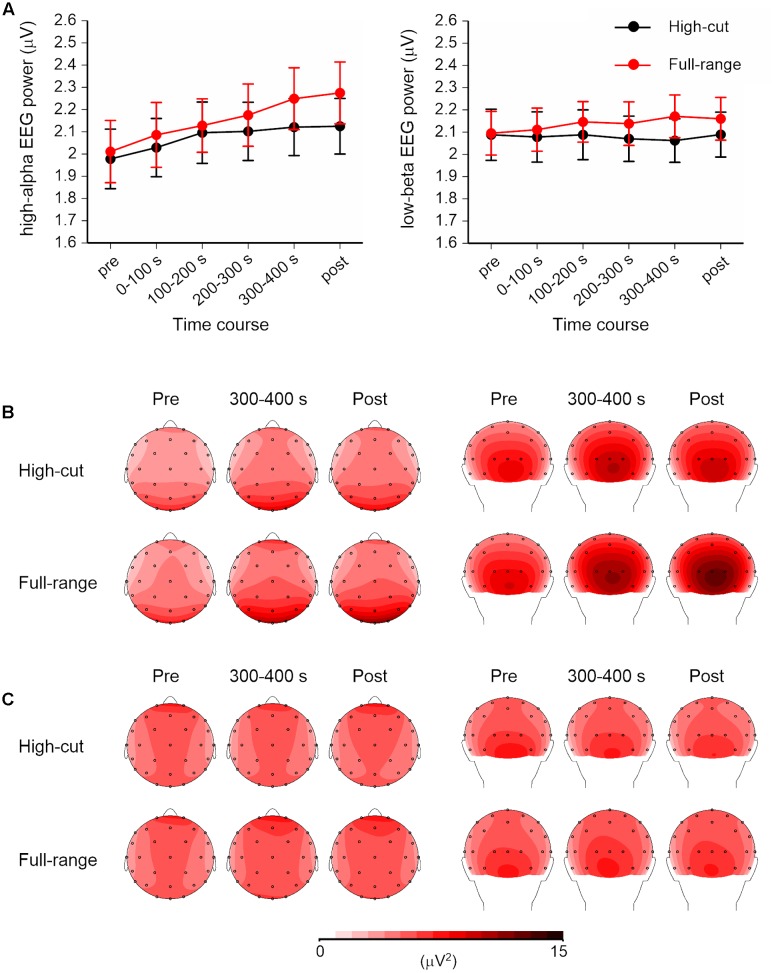
**High-alpha (10.5–13 Hz) and low-beta (13–20 Hz) EEG powers for the full-range and high-cut conditions.** **(A)** Time course of the square root of EEG powers over four scalp regions. Error bars show SEs. No music was played in the pre- and post-music epochs. **(B,C)** Scalp topography of the high-alpha and low-beta EEG powers in the pre-music, 300–400 s (i.e., last quarter of the listening period), and post-music epochs. Left: top view. Right: back view.

**Figure 2** shows the time course and scalp topography of high-alpha EEG (10.5–13 Hz) and low-beta EEG (13–20 Hz) bands. For EEG measures, a Sound Type × Epoch × Anterior-Posterior × Hemisphere ANOVA was conducted for each frequency band. Significant effects of sound type were found for both bands. For other frequency bands, only the theta EEG band (4-8 Hz) power showed a significant Sound Type × Anterior-Posterior × Hemisphere interaction, *F*(1,21) = 5.37, *p* = 0.031, $\eta_{p}^{2}$ = 0.20. However, no significant simple main effects were found.

For high-alpha EEG band, the Sound Type × Epoch × Hemisphere interaction was significant, *F*(5,17) = 7.06, *p* = 0.001, $\eta_{p}^{2}$ = 0.67. Separate ANOVAs for each epoch revealed a significant Sound Type × Hemisphere interaction at the 200-300-s epoch, *F*(1,21) = 12.63, *p* = 0.002, $\eta_{p}^{2}$ = 0.38, and a significant effect of sound type at the post-music period, *F*(1,21) = 6.99, *p* = 0.015, $\eta_{p}^{2}$ = 0.25. *Post hoc* tests revealed that high-alpha EEG power was greater for the full-range excerpt than for the high-cut excerpt and that the sound type effect was found for the left but not right hemisphere at the 200-300-s epoch. No effects of sound type were obtained at the epochs before 200 s. The main effect of anterior-posterior was also significant, *F*(1,21) = 15.67, *p* = 0.001, $\eta_{p}^{2}$ = 0.43, showing that the high-alpha EEG was dominant over posterior scalp sites.

For low-beta EEG band, the Sound Type × Anterior-Posterior × Hemisphere interaction and the main effect of sound type effect were significant, *F*(1,21) = 4.49, *p* = 0.046, $\eta_{p}^{2}$ = 0.18; *F*(1,21) = 5.43, *p* = 0.030, $\eta_{p}^{2}$ = 0.21. Low-beta EEG power was greater in the full-range condition than in the high-cut condition. Separate ANOVAs for anterior-posterior and hemisphere also revealed significant effects of sound type, for posterior region: *F*(1,21) = 7.07, *p* = 0.015, $\eta_{p}^{2}$ = 0.25; for left hemisphere: *F*(1,21) = 5.26, *p* = 0.032, $\eta_{p}^{2}$ = 0.20; for right hemisphere: *F*(1,21) = 5.27, *p* = 0.032, $\eta_{p}^{2}$ = 0.20; except for anterior region: *F*(1,21) = 3.94, *p* = 0.060, $\eta_{p}^{2}$ = 0.16. Although there were no significant interaction effects including epoch, **Figure 2** shows that the difference between the full-range and high-cut excerpts seems to be more prominent at later epochs. Two-tailed *t*-tests revealed significant differences between the two excerpts at the 200-300-s, 300-400-s, and post epochs, *t*s(21) > 2.37, *p*s < 0.027; *p* > 0.114 at the epochs before 200 s.

### Grand Mean ERPs for the Visual Vigilance Task

**FIGURE 3:**
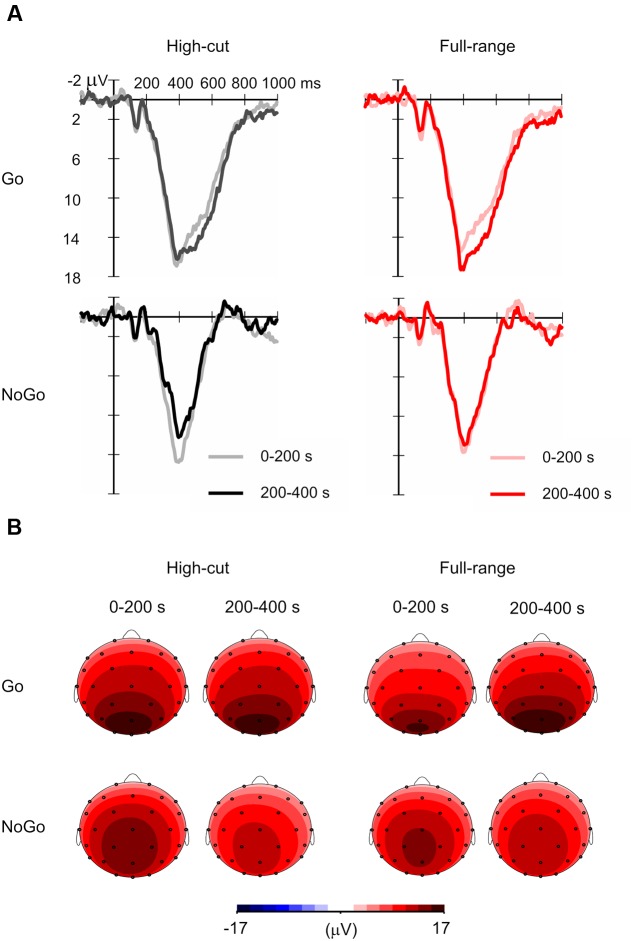
**Grand mean event-related potential (ERP) waveforms for Go and NoGo stimuli in the full-range and high-cut conditions.** **(A)** Waveforms at Pz. **(B)** Scalp topography of P3 amplitudes in the 0–200 s (i.e., first half) and 200–400 s (i.e., second half) epochs of music listening. The mean amplitudes of 380–440 ms after stimulus onset in the grand mean ERP waveforms are shown.

**Figure 3** shows grand mean ERP waveforms and the scalp topography of the Go and NoGo P3 amplitudes. The mean number of averaged trials was 18.6 (range = 13-20). Although this is less than an optimal number of averages for P3 (Cohen and Polich, 1997), P3 peaks can be detected for all individual ERP waveforms. **Table 1** shows the mean amplitudes and latencies of the P3 peaks.

**Table 1:** The peak amplitudes and latencies of the P3 component at Pz.

		High-cut		Full-range	
			
		0–200 s	200–400 s	0–200 s	200–400 s
Go	Amplitude (μV)	18.3 (6.4)	18.0 (5.7)	16.7 (6.3)	18.6 (6.3)
	Latency (ms)	404.9 (37.2)	419.5 (42.2)	405.1 (34.8)	420.2 (41.7)
No Go	Amplitude (μV)	16.3 (6.6)	13.1 (5.3)	15.1 (6.9)	14.0 (7.1)
	Latency (ms)	408.0 (35.0)	412.2 (35.7)	417.8 (41.2)	413.5 (27.9)


*Values in brackets are *SD.**

For the P3 amplitude, a Sound Type × Epoch ANOVA was conducted for Go and NoGo stimulus conditions separately. A significant interaction was found for the Go condition, *F*(1,21) = 4.39, *p* = 0.049, $\eta_{p}^{2}$ = 0.17, but not for the NoGo condition, *F*(1,21) = 2.64, *p* = 0.119, $\eta_{p}^{2}$ = 0.11. *Post hoc* tests revealed that Go P3 amplitude increased from the 0-200 s to the 200-400 s epoch for the full-range excerpt, whereas Go P3 amplitude did not change for the high-cut excerpt. The main effect of epoch was significant for the NoGo condition, *F*(1,21) = 13.39, *p* = 0.001, $\eta_{p}^{2}$ = 0.39, showing that NoGo P3 amplitude decreased during the task for both musical excerpts.

Similar ANOVAs were conducted for latencies. No significant main or interaction effects of sound type were found. The main effect of epoch was significant for the Go stimulus condition, *F*(1,21) = 5.01, *p* = 0.036, $\eta_{p}^{2}$ = 0.19, showing that Go P3 latency increased through the task.

One of the reviewers questioned about the effects of sound type on the Nogo N2 (Falkenstein et al., 1999). We conducted a Sound Type × Epoch ANOVA on the amplitude of the Nogo N2 (Nogo minus Go in the 200–300 ms period at Fz and Cz). No significant main or interaction effects were found.

### Behavioral and Other Physiological Measures

**FIGURE 4:**
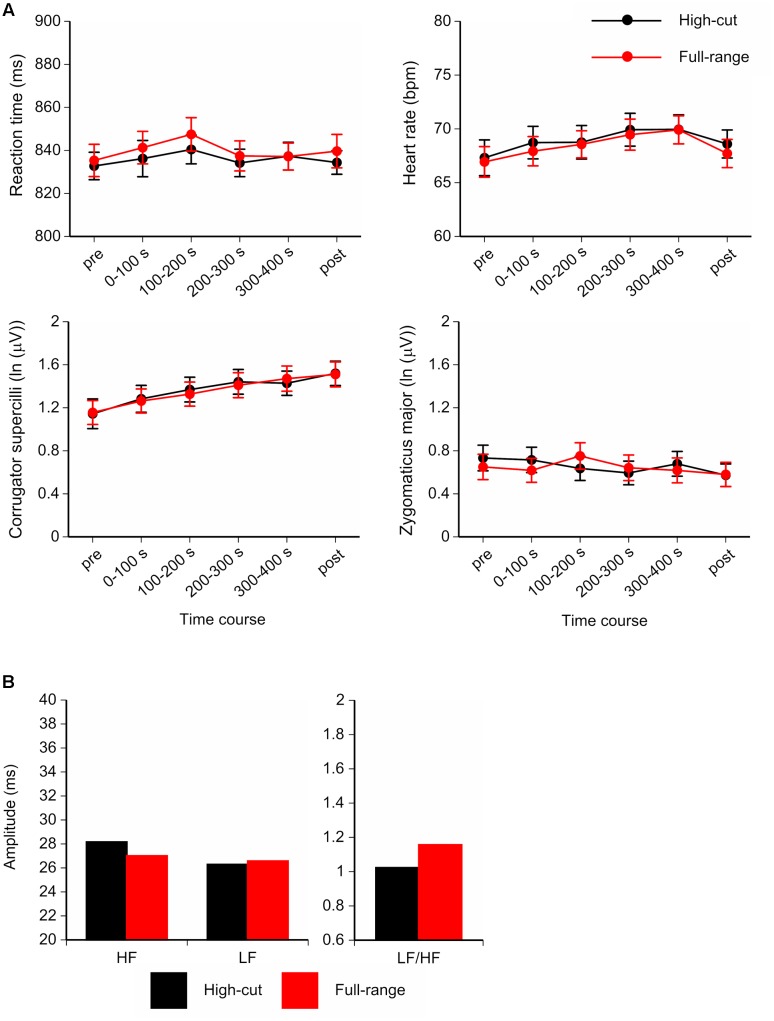
**(A)** Time course of the mean Go reaction times, Heart rate (HR), and facial electromyograms (EMGs; corrugator supercilii and zygomaticus major) for the full-range and high-cut conditions. **(B)** Amplitudes and ratio of heart rate variability (HRV) components for the last 300-s epoch of musical excerpts. Error bars show SEs.

Participants performed the vigilance task with considerable accuracy (high-cut: *M* = 98.6%, 95.8-100%; full-range: *M* = 97.9%, 95.0-99.2%). **Figure 4** shows the time course of mean Go reaction times, HR, and facial EMGs (corrugator supercilii, zygomaticus major), and the HRV components for the last 300-s epoch of the musical excerpts. For the corrugator supercilii, a Sound Type × Epoch ANOVA showed a significant main effect of epoch, *F*(5,17) = 5.69, *p* = 0.003, $\eta_{p}^{2}$ = 0.63. Corrugator activity increased over the course of the task. No significant main or interaction effects of sound type were found for RT or other physiological measures.

### Subjective Ratings

**FIGURE 5:**
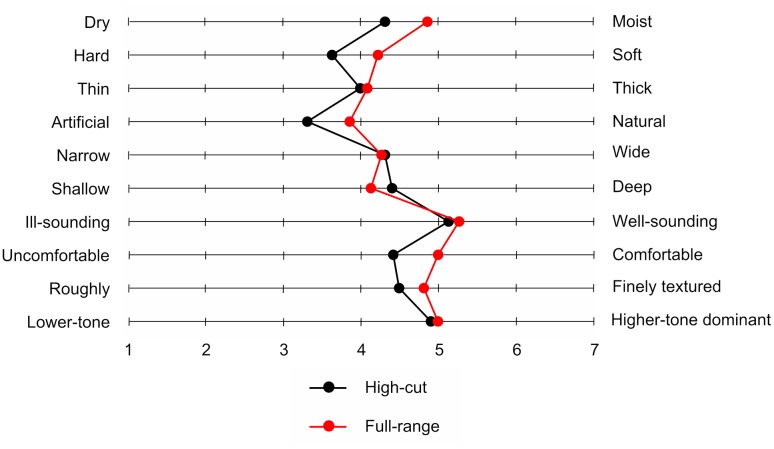
**Mean sound quality ratings for two musical excerpts with or without inaudible high-frequency components**.

**Table 2** shows mean scores for participants’ mood states. A significant difference between the two types of musical excerpt was found only for inactive pleasantness scores, *t*(21) = 3.13, *p* = 0.005. Participants provided higher inactive pleasantness scores under the full-range than under the high-cut excerpt. **Figure 5** shows the mean sound quality ratings for the full-range and high-cut musical excerpts. No significant differences were found between the two types of audio source for any adjective pairs, *t*s(21) < 1.92, *p*s > 0.069. The correct rate of the forced choices was 41.0%, which did not exceed chance level (*p* = 0.523, binomial test).

**Table 2:** Mean scores and the results of two-tailed *t*-tests for participants’ mood states.

	High-cut	Full-range	*t*(21)	*p*
Affect Grid (9-point scale, 1–9)				
Pleasantness	6.3 (1.4)	6.4 (1.0)	0.32	0.754
Arousal	4.9 (2.3)	4.4 (2.1)	1.15	0.264
Multiple mood scale (4-point scale, 1–4)				
Depression	1.5 (0.5)	1.5 (0.5)	0.00	1.00
Aggression	1.1 (0.3)	1.1 (0.3)	0.40	0.690
Fatigue	1.9 (0.6)	1.8 (0.5)	1.27	0.219
Active pleasantness	1.8 (0.6)	1.8 (0.5)	0.09	0.929
Inactive pleasantness	2.7 (0.6)	3.0 (0.6)	3.13	0.005
Affinity	1.7 (0.6)	1.7 (0.7)	0.66	0.518
Concentration	2.1 (0.5)	2.2 (0.6)	1.50	0.148
Startle	1.5 (0.5)	1.4 (0.5)	0.86	0.400


*Values in brackets are *SD.**

## Discussion

High-resolution audio with inaudible high-frequency components is a closer replication of real sounds than similar and indistinguishable sounds in which these components are artificially cut off. It remains unclear what kind of advantages high-resolution audio might have for human beings. Previous research in which participants listened to high-resolution music under resting conditions have shown that alpha and low-beta EEG powers were larger for an excerpt with high-frequency components as compared with an excerpt without them (Oohashi et al., 2000, 2006; Yagi et al., 2003a; Fukushima et al., 2014; Kuribayashi et al., 2014; Ito et al., 2016). The present study asked participants to listen to two types of high-resolution audio of the same musical piece (with or without inaudible high-frequency components) while performing a vigilance task in the visual modality. Although the effect size is small, the overall results support the view that the effect of high-resolution audio with inaudible high-frequency components on brain activity reflects a relaxed attentional state without conscious awareness.

We found greater high-alpha (10.5–13 Hz) and low-beta (13–20 Hz) EEG powers for the excerpt with high-frequency components as compared with the excerpt without them. The effect appeared in the latter half of the listening period (200-400 s) and during the 100-s period after music presentation (post-music epoch). Furthermore, for full-range sounds compared with high-cut sounds, Go trial P3 amplitude increased, and subjective relaxation scores were greater. Because task performance did not change across musical excerpts, with no difference in self-reported arousal, the effects of high-resolution audio with inaudible high-frequency components on brain activities should not reflect a decrease of listeners’ arousal level. These findings show that listeners seem to experience a relaxed attentional state when listening to high-resolution audio with inaudible high-frequency components compared to similar sounds without these components.

It has been shown that listening to musical pieces increases EEG powers of theta, alpha, and beta bands (Pavlygina et al., 2004; Jäncke et al., 2015), and that the enhanced alpha-band power holds for approximately 100 s after listening (Sanyal et al., 2013). Therefore, high-resolution audio with inaudible high-frequency components would be advantageous compared to a similar digital audio in which these components are removed, in terms of the enhanced brain activity. Kuribayashi and Nittono (2014) have localized the intracerebral sources of this alpha EEG effect using standardized low-resolution brain electromagnetic tomography (sLORETA). The analysis revealed that the difference between full-range and high-cut sounds appeared in the right inferior temporal cortex, whereas the main source of the alpha-band activity was located in the parietal-occipital region. The finding that the alpha-band activity difference was obtained in specific but not whole regions is suggestive that this increase may reflect an activity related to task performance rather than a global arousal effect (Barry et al., 2007).

The present study shows that not only high-alpha and low-beta EEG powers but also P3 amplitude increased in the last half of the listening period (200-400 s). Alpha-band EEG activity and P3 amplitude have been shown to be positively correlated, in such a way that prestimulus alpha directly modulates positive potential amplitude in an auditory equiprobable Go/NoGo task (Barry et al., 2000; De Blasio et al., 2013). P3 amplitude is larger when greater attentional resources are allocated to the eliciting stimulus (Kok, 1997, 2001; Polich, 2007). Alpha power is increased in tasks requiring a relaxed attentional state such as mindfulness and imagination of music (Cooper et al., 2006; Schaefer et al., 2011; Lomas et al., 2015). Increased alpha power is thought to be a signifier of enhanced processing, with attention focused on internally generated stimuli (Lomas et al., 2015). Beta power has been shown to increase when arousal and vigilance level increase (e.g., Sebastiani et al., 2003; Aftanas et al., 2006; Gola et al., 2012; Kamiñski et al., 2012). Taken together, the EEG and ERP results support the idea that listening to high-resolution audio with inaudible high-frequency components enhances the cortical activity related to the attention allocated to task-relevant stimuli. Although the effect was not observed in behavior, the gap between behavioral and EEG and ERP results is probably due to the ceiling effect of the vigilance task performance. Such a gap is often observed in other studies. For example, Okamoto and Nakagawa (2016) similarly reported that event-related synchronization in the alpha band during working memory task was increased 20–30 min after the onset of the exposure to blue (short-wavelength) light, as compared with green (middle-wavelength) light, while task performance was high irrespective of light colors.

As a mechanism underlying the effect of inaudible high-frequency sound components, we speculate that the brain may subconsciously recognize high-resolution audio that retains high-frequency components as being more natural, as compared with similar sounds in which such components are artificially removed. A link between alpha power and ratings of ‘naturalness’ of music has been reported. When listening to the same musical piece with different tempos, alpha-band EEG power increased for excerpts that were rated to be more natural, the ratings of which were not directly related to subjective arousal (Ma et al., 2012; Tian et al., 2013). As high-resolution audio replicates real sound waves more closely, it may sound more natural (at least on a subconscious level) and facilitate music-related psychophysiological responses.

Our findings have some limitations. First, because we used only a visual vigilance task, it is unclear whether high-resolution audio can improve performance on tasks that involve working memory and long-term memory. Because a vigilance task is relatively easy, our participants were able to sustain high performance. Other research using an n-back task requiring memory has shown that high-resolution audio also enhances task performance (Suzuki, 2013). Future research will benefit from using other tasks requiring various cognitive domains and processes.

Second, the underlying mechanism of how inaudible high-frequency components affect EEG activities cannot be revealed by the current data. It is noteworthy that presenting high-frequency components above 20 kHz alone did not produce any change in EEG activities (Oohashi et al., 2000). Therefore, the combination of inaudible high-frequency components and audible low-frequency components should be a key factor that causes this phenomenon. A possible clue was obtained by a recent study of Kuribayashi and Nittono (2015). Recording sound spectra of various musical instruments, they found that high-frequency components above 20 kHz appear abundantly during the rising period of a sound wave (i.e., from the silence to the maximal intensity, usually less than 0.1 s), but occur much less after that. Artificially cutting off the high-frequency components may cause a subtle distortion in this short period. It will take some time to accumulate these small, short-lasting differences until they produce discernible psychophysiological effects. This explanation is consistent with the fact that the effect of high-frequency components on EEG activities appears only after a 100–200-s exposure to the music (Oohashi et al., 2000, 2006; Yagi et al., 2003a; Fukushima et al., 2014; Kuribayashi et al., 2014; Ito et al., 2016).

Third, it remains unclear why there was a time lag until the effects of high-resolution audio on brain activity show up, and why this effect was maintained for 100 s after music stopped. A possible reason is that, as mentioned above, sufficiently long exposure is needed for the effects of inaudible high-frequency components. Another possibility is that listening to music has psychophysiological impact through the engagement of various neurochemical systems (Chanda and Levitin, 2013). Humoral effects are characterized by slow and durable responses, which might be underlying the lagged effect of high-resolution audio with inaudible high-frequency components. Although the present study did not reveal this effect on autonomic nervous system (HR and HRV) indices during music listening, participants reported greater relaxation scores after listening to high-resolution music with inaudible high-frequency components. It is a task for future research to determine the time course of the effect more precisely.

Fourth, the present study did not manipulate the sampling frequency and the bit depth of digital audio. High-resolution audio is characterized not only by the capability of reproducing inaudible high-frequency components but also by more accurate sampling and quantization (i.e., a higher sampling frequency and a greater bit depth) as compared with low-resolution audio. If the naturalness derived by a closer replication of real sounds affects EEG activities, the sampling frequency and the bit depth would do too regardless of whether the real sounds feature high-frequency components. This idea would be worth examining in future research.

In summary, high-resolution audio with inaudible high-frequency components has some advantages over similar and indistinguishable sounds in which these components are artificially cut off, such that the former type of digital audio induces a relaxed attentional state. Even without conscious awareness, a closer replication of real sounds in terms of frequency structure appears to bring out greater potential effects of music on human psychophysiological state and behavior.

## Ethics Statement

This study was carried out in accordance with the recommendations of The Research Ethics Committee of the Graduate School of Integrated Arts and Sciences in Hiroshima University. All participants gave written informed consent in accordance with the Declaration of Helsinki. The protocol was approved by The Research Ethics Committee of the Graduate School of Integrated Arts and Sciences in Hiroshima University.

## Author Contributions

RK and HN planned the experiment, interpreted the data, and wrote the paper. RK collected and analyzed the data.

## Conflict of Interest Statement

The authors declare that the research was conducted in the absence of any commercial or financial relationships that could be construed as a potential conflict of interest.
